# Supplemented Feed for Broiler Chickens: The Influence of Red Grape Pomace and Grape Seed Flours on Meat Characteristics

**DOI:** 10.3390/ani16020280

**Published:** 2026-01-16

**Authors:** Manuela Mauro, Alessandro Attanzio, Carla Buzzanca, Marialetizia Ponte, Vita Di Stefano, Ignazio Restivo, Giuseppe Maniaci, Angela D’Amico, Antonino Di Grigoli, Emiliano Gurrieri, Antonio Fabbrizio, Sabrina Sallemi, Luisa Tesoriere, Francesco Longo, Rosario Badalamenti, Aiti Vizzini, Maria Grazia Cappai, Mirella Vazzana, Vincenzo Arizza

**Affiliations:** 1 Department of Biological, Chemical and Pharmaceutical Sciences and Technologies (STEBICEF), University of Palermo, 90123 Palermo, Italy; manuela.mauro01@unipa.it (M.M.); alessandro.attanzio@unipa.it (A.A.); carla.buzzanca@unipa.it (C.B.); ignazio.restivo@unipa.it (I.R.); angela.damico02@unipa.it (A.D.); luisa.tesoriere@unipa.it (L.T.); francesco.longo03@unipa.it (F.L.); rosario.badalamenti@unipa.it (R.B.); aiti.vizzini@unipa.it (A.V.); mirella.vazzana@unipa.it (M.V.); vincenzo.arizza@unipa.it (V.A.); 2 Dipartimento di Scienze Agrarie, Alimentari e Forestali (SAAF), University of Palermo, Viale delle Scienze Ed.4, 90128 Palermo, Italy; giuseppe.maniaci@unipa.it (G.M.); antonino.digrigoli@unipa.it (A.D.G.); 3 Independent Researcher, 97015 Modica, Italy; emilianogurrieri@gmail.com (E.G.); medvet80@hotmail.it (S.S.); 4 Facoltà di Ingegneria, Università eCampus, Via Isimbardi, 22060 Novedrate, Italy; antfab13@gmail.com; 5 Dipartimento di Medicina Veterinaria, University of Sassari, Via Vienna n. 2, 07100 Sassari, Italy; mgcappai@uniss.it

**Keywords:** wine waste, bioactive molecules, chicken meat, antioxidant, radical scavenging activity, fatty acids composition, total polyphenolic content

## Abstract

Intensive broiler chicken farming is one of the most important livestock sectors globally. However, intensive production systems raise concerns about farm sustainability, as well as ensuring animal welfare and product quality. For this reason, identifying novel, high-value-added feed ingredients is crucial. Winery by-products (WBPs) are a valuable source of bioactive compounds and can be utilized as functional feed ingredients. This study evaluated the effects of dietary supplementation with grape seed meal and grape pomace meal in diets for broilers up to 42 days of age. Three dietary treatments were formulated—grape seed meal (3% and 6%), grape pomace meal (3% and 6%), and a combination (3% seed meal + 3% pomace meal)—along with a standard diet (control). The proximal composition (moisture, protein, fatty acid profile, fats, ash), antioxidant parameters (ROS, GSH, NO, POV), free radical scavenging activity (*DPPH* and *ABTS^•+^*), and total phenolic content of the meat and physical characteristics (color) were assessed. While proximal composition of meat was not significantly influenced by the dietary treatment, some parameters, such as total phenolic content, PUFA levels, and antioxidant and free radical scavenging activity, were improved. These results demonstrate enhanced favorable traits improving chicken meat quality and confirm the potential of WBPs as functional feed ingredients, promoting a more sustainable production model aligned with the principles of the circular economy.

## Simple Summary

Intensive broiler chicken farming requires innovative strategies to improve product quality and sustainability. Winery by-products (WBPs) are a promising source of bioactive compounds for feed inclusion. This study evaluated the impact on meat quality of supplementing broiler diets with grape seed meal and/or grape pomace meal, individually (at 3% or 6%) or in combination (3% + 3%). The meat’s nutritional profile, total phenolic content, and antioxidant/scavenging capacities were measured. The results showed that, although the basic nutritional composition remained stable, the diet containing the combination of 3% pomace meal and 3% seed meal yielded the most significant improvements: increased phenolic content, higher PUFA levels, and enhanced antioxidant and radical scavenging activity. Therefore, the synergistic inclusion of WBPs emerges as an effective strategy for functionally enriching chicken meat, while simultaneously supporting a production model based on the principles of the circular economy.

## 1. Introduction

The global demand for broiler chicken meat continues to grow, leading to the consolidation of this livestock sector due to high production efficiency; however, at the same time, concerns have been raised regarding animal welfare and the nutritional quality of the final product [1,2]. Given how much chicken meat is inherently valued for its favorable profile (low fat and cholesterol, high protein content, and high concentrations of polyunsaturated fatty acids, PUFAs) [3,4], research has been intensely oriented toward optimizing feed performance and safely employing by-products with biologically active compounds, with the aim of finding alternative solutions to antibiotic growth promoters (AGPs) as functional feed additives [5,6,7]. In this scenario, there is a strong drive toward using natural ingredients that can strengthen the intestinal and immune health of animals, thereby supporting safer and more sustainable poultry production [8,9,10,11]. At the same time, the regulatory framework, especially within the European Union, has emphasized the transition toward a circular economy, where industrial waste must be actively valorized rather than simply disposed of, transforming post-production residues into valuable resources. It is within this context that winery by-products (WBPs) emerge as ideal candidates, originating from one of the world’s major agri-food chains [12]. These wastes—pomace (GP), seeds (GSs), and rachis—are known to contain concentrates of highly beneficial bioactive molecules including fiber, protein, minerals, and, most importantly, polyphenols (such as anthocyanins and tannins) that confer potent antioxidant, antibacterial, and antiviral properties [13,14]. Specifically, GSs are distinguished by their high concentration of PUFAs and vitamin E, which are essential for cellular protection and significantly improve the meat’s lipid profile [15]. GP, on the other hand, serves as a primary source of phenolic compounds and dietary fiber [16,17,18]. The inclusion of these components has been positively linked to improving the fatty acid profile of chicken meat, specifically by increasing the beneficial n-3 fraction and reducing saturated fats. Furthermore, while the beneficial effects of individual WBP components have been established, preliminary studies suggest that the protective effect against lipid oxidation is synergistic when GP and GS are combined rather than used in isolation [19]. Despite these clear advantages, the effectiveness of WBP supplementation is strictly dose-dependent; excessive inclusion must be avoided as high tannin content can negatively affect feed palatability and hinder the absorption of proteins and iron [20].

In this regard, the literature shows contrasting optimal inclusion rates of grape by-products: certain levels (<3%) are recommended for growth [21], while others (up to 6%) favor meat quality [22]. Therefore, optimizing the inclusion level and method (single vs. combined) remains a critical research gap that needs to be addressed to maximize the functional and nutritional transfer of WBP components to the final product. This study addresses two aspects: the direct comparison of defatted GS meal vs. grape skin meal within the same trial, and the evaluation of the dose–response (3% and 6%) to find the optimal trade-off between performance and quality in PUFA-enriched diets. These levels were chosen as lower and upper thresholds to explore the variability between the two raw materials. In a previous study [23] using the same experimental design, we already observed a significant improvement in esterase, alkaline phosphatase, and peroxidase activity, as well as blood glucose levels, reactive oxygen species (ROS), and glutathione (GSH), with particularly marked variations after 21 and 42 days of administration. Furthermore, all treatments showed effects that were dependent on both the exposure time and the concentration of the by-product, with a more evident response in diets containing GS meal [23]. The controlled inclusion of WPBs, particularly GP and GSs, in broiler chicken diets could prove to be an effective strategy to improve not only the antioxidant profile of meat but also its nutritional value. This strategy also helps promote a more sustainable livestock production model, aligned with the principles of the circular economy, without compromising animal health or production performance. Therefore, this study aims to define the optimal level at which these by-products (GP and GS) should be included in diets to ensure their safe and effective use.

## 2. Materials and Methods

### 2.1. Wine Waste Flours

Two types of flour, grape seed flour (GSF) and grape pomace flour (PMF), were produced as described in our previous studies [23,24] from WBPs derived from the processing of Sangiovese red wine cultivated in western Sicily (Italy) under organic farming conditions and characterized by high sun exposure, mild temperatures, and moderate ventilation (September 2020). The residues were initially sieved to separate the grape seeds and pomace. For GSF, the grape seeds were dried at 24 °C for 4 days, pressed (Cgoldenwall CAN-684, Hangzhou, China) to remove their oil, and subsequently ground. For PMF, the grape pomace was dried at 55 °C for two days and ground directly.

The flours obtained were subjected to chemical analysis according to the AOAC (2005) methods [25], the results of which have been published by us in a previous study [23].

### 2.2. Experimental Plan: Animals and Diet

A total of 240 one-day-old chicks (Ross 308 strain, Aviagen, Cocconato, Italy) were included in a 42-day feeding trial at the Leocata Mangimi S.p.A. facilities, adhering to the maximum stocking density established by European Directive 2007/43/EC [26]. To ensure uniformity, only male subjects were used for all measurements. The animals were housed on litter (wood shavings) in pens measuring 1.2 m × 1.2 m, with a regulated photoperiod (23 h light/1 h dark for the first 7 days, then 20 h light/4 h dark) and controlled temperature (32 °C for the first week, subsequently reduced to 26 °C). Water lines were disinfected before the start of the study [27]. Three separate trials were conducted, for a total of 8 experimental groups, to test supplementation with grape pomace flour (P), grape seed flour (G), and their combination. Each experimental group consisted of 30 birds, each further divided into two replicates of 15 subjects.

In Trial 1, a total of 90 birds were used, with treatments P0 (0% grape pomace), P3 (3% grape pomace), and P6 (6% grape pomace).

In Trial 2, a total of 90 birds were used, with treatments G0 (0% grape seed), G3 (3% grape seed), and G6 (6% grape seed).

In Trial 3, a total of 60 birds were used, with treatments P0G0 (0% grape pomace and grape seed) and P3G3 (3% grape pomace and 3% grape seed).

The animals were fed according to the nutritional specifications suggested by a genetics company [28], and each group received four different diets during their growth stages: pre-starter diets (1 to 11 days), starter diets (11 to 24 days), grower diets (25 to 39 days), and finisher diets (40 days to slaughter). A maximum inclusion rate of 6% was chosen to balance each diet; all diets were formulated to be isocaloric and comparable to the standard diet in terms of amino acids and vitamins. The chemical composition of grape pomace and grape seed meal, as well as the nutritional composition of the four different diets, has been previously reported in our study using the same experimental design to evaluate other parameters [23] (see Supplementary Materials). Growth was monitored by individual weighing at 7, 21, and 42 days to calculate the average daily gain (ADG); these data have already been reported in a previous study [23]. On day 42, four broiler chickens per group were randomly selected and slaughtered by cervical dislocation (EU Regulation 1099/2009) [29]. Following slaughter, muscle tissue samples (*pectoralis major*) were collected and weighed for lyophilization and subsequent analyses [23].

### 2.3. Physico-Chemical Properties of Broiler

Physico-chemical properties were determined for samples of muscle tissue. All analyses were performed in triplicate. The color of the meat was determined for the muscle samples immediately after slaughter. Colorimetric evaluation was conducted using a Chroma Meter (CR300, Minolta Corporation Ltd., Osaka, Japan) calibrated against the white standard L* = 100 (BaSO_4_) and operating with Illuminant C and an aperture size of ø 8 mm. Results were expressed in the L*, a*, and b* system indicating perceptual lightness (L*), redness/greenness (a*), and yellowness/blueness (b*). Then, Warner–Bratzler shear force (WBS) was measured using an Instron Universal Testing Machine (Instron tester 5564, Trezzano sul Naviglio, Milan, Italy) equipped with the appropriate device. For this determination, three sub-samples were prepared for each raw muscle sample, with standardized dimensions of 12.5 mm in diameter and 25 mm in length. Subsequently, the muscle samples were packaged, and weighed before being frozen at −80 °C. Samples were then unfrozen for analysis. Moisture, protein, fat, and ash contents were quantified according to the official AOAC methods (AOAC, 2012) [25], with nitrogen converted to protein using a conversion factor of 6.25.

### 2.4. ROS, GSH, NO, and POV Evaluation

#### 2.4.1. Sample Preparation

To determine the levels of reactive oxygen species (ROS), glutathione (GSH), and nitric oxide (NO), 30 mg of muscle tissue was homogenized in 3 mL of cold PBS1X. This homogenization was performed while maintaining a precise mass-to-volume ratio of 1:100.

#### 2.4.2. Fluorescence Detection of GSH and ROS in Muscle Tissue

Quantification of ROS and GSH was performed using fluorescent dyes, adapting a previously established protocol for broiler plasma samples [23], with the methodological variations described. Analyses were conducted using the Promega™ GloMax^®^ Plate Reader (Milan, Italy) set to an excitation wavelength of 485 nm and an emission wavelength of 530 nm. Each sample was measured in parallel and in triplicate, subtracting blank and autofluorescence controls. GSH content was assessed using CellTracker Green CMFDA dye (Thermo Fisher Scientific, Waltham, MA, USA, Cat. No. C2925). The reaction mixture consisted of 4 µL of muscle homogenate, 96 µL of phosphate buffer (0.1 M pH 7.2), and 5 µL of CellTracker Green CMFDA (10 μM in DMSO). ROS levels were determined with the CM-H2DCFDA dye (Thermo Fisher Scientific, Waltham, MA, USA, Cat. No. c6827) by mixing 10 µL of muscle homogenate, 85 µL of phosphate buffer (0.1 M pH 7.2), and 10 µL of CM-H2DCFDA (10 μM in DMSO). In both assays, the blank control was free of homogenate and the autofluorescence control was free of dye. Fluorescence was measured after a 30 min incubation period in the dark at room temperature. Finally, all detected fluorescence values were normalized to the protein content of each sample, determined by the Bradford assay.

#### 2.4.3. Determination of Nitric Oxide

NO levels in muscle tissue were determined colorimetrically using the Griess reaction (Thermo Fisher Scientific Inc., Waltham, MA, USA). For analysis, 100 µL of the supernatant—obtained after centrifugation at 14,000 rpm for 30 min at 4 °C—was incubated with an equal volume of Griess reagent (1% sulfanilamide in 5% phosphoric acid and 0.1% N-(1-naphthyl)-ethylenediamine). After a 10 min incubation period, absorbance was read spectrophotometrically at 520 nm using a GloMax^®^ plate reader (Promega Italia Srl, Milano, Italy).

#### 2.4.4. Determination of Peroxide Value

The oxidative status of meat fat was assessed by measuring the peroxide value (POV), expressed as mEq O_2_/kg of fat, in triplicate according to the IDF protocol (1991) [30] in order to determine the primary lipid oxidation index.

### 2.5. Fatty Acid Analysis

Fatty acid analysis of freeze-dried muscle tissue was performed in triplicate as reported previously [31]. Two grams (2 g) of each dried sample was extracted with 10 mL of CHCl3/MeOH (2:1) and sonicated for 50 min at 60 °C, followed by cooling to room temperature and centrifugation (4000× *g* for 20 min). The pellet was subjected to three additional extractions. Water-soluble compounds were removed by separation with H_2_O/NaCl solution (0.88%), and the resulting lipid solution was evaporated. The oily residue was resuspended in 1 mL of hexane, transesterified by adding 100 µL of KOH/MeOH (2M), and vortexed for 3 min. For gas chromatography–mass spectrometry (GC/MS) analysis (Thermo Fisher, Waltham, MA, USA), a mixture of 100 µL of the hexane phase (FAMEs), 100 µL of the internal standard (ethyl myristate 150 ppm), and 800 µL of hexane was prepared. The determination was performed with an ISQ™ 9000 quadrupole GC-MS system (Thermo Fisher Scientific, Waltham, MA, USA) and a ZB-WAX^®^ column (30 m × 0.25 mm × 0.25 μm, film thickness; Phenomenex, Bologna, Italy). Helium (1 mL/min) was used as the carrier gas, with an injection at 210 °C and an injection volume of 5 µL (split ratio 10:1). The oven thermal program started at 130 °C for 3 min, increasing to 280 °C with a ramp of 1.5 °C/min, and then to 350 °C with a ramp of 10 °C/min (6 min isotherm). FAMEs were identified by comparing retention times and MS spectra with the external standard (Supelco 37 Component FAME Mix, Milan, Italy) and the NIST 2011 database. Integration was managed with XcaliburTM 4.3 software (Thermo ScientificTM, Waltham, MA, USA). FAME quantification was expressed as relative percentage.

### 2.6. Evaluation of Total Phenolic Content and Radical Scavenging Activity

Total phenolic content (TPC) was determined using the optimized Folin–Ciocâlteu colorimetric method [32], with minor modifications: 1 g of muscle tissue was mixed with 12 mL of methanol/water (80:20 *v*/*v*), sonicated for 45 min, and filtered (PTFE 0.45 μm). The filtrate aliquot (0.120 mL) was incubated with 120 μL of Na_2_CO_3_ solution and 625 μL of Folin–Ciocalteu reagent (1:5) in the dark at 25 °C for 60 min. Absorbance was evaluated at λ = 765 nm (Varian Cary 50 spectrophotometer, Agilent Technologies, Milano, Italy); methanol was used as a blank, and the calibration curve was constructed with gallic acid (GA) (0.001 to 0.25 mg/mL). TPC was expressed as mg GA equivalents per g (mg GAE × g^−1^) of sample. Radical scavenging activity was evaluated using the DPPH and ABTS^•+^ assays [33,34].

For the DPPH assay, 1 g of sample was extracted with 8 mL of methanol, sonicated, and filtered. Then, 100 µL of the filtrate was mixed with 3 mL of DPPH (60 µM) and incubated in the dark at 25 °C for 30 min, with methanol as a blank; the activity was measured at λ = 515 nm.

For the ABTS^•+^ assay, a 2,2′-azinobis (3-ethylbenzothiazoline-6-sulfonic acid) (ABTS)^•+^ radical cation was prepared following a previously described method by reacting ABTS^•+^ with potassium persulfate [35]. Then, 2 g of sample was extracted with 4 mL of MeOH, sonicated, and filtered; absorbance was read at λ = 734 nm 5 min after the addition of 3 mL of diluted ABTS^•+^ to 100 µL of sample, using ethanol as a reference. For both antioxidant assays, results were reported as Trolox equivalent antioxidant activity (TEAC), expressed as mmol Trolox equivalents (TE) per 100 g of sample using a Trolox calibration curve [2.5 µM–30 µM]. All data were run in triplicate and reported as the mean ± standard error of the mean (SEM).

### 2.7. Statistical Analysis

The analysis was performed using Prism 10.1.2 software (GraphPad Software Inc., San Diego, CA, USA). The data from the three different experimental trials were analyzed separately, using a one-way model including the effect of diet. The Tukey multiple comparison test was used for mean comparisons within each trial. The significance threshold was set at *p* < 0.05.

## 3. Results

### 3.1. Physico-Chemical Properties of Broiler Chicken Muscle Tissue Samples

Dietary supplementation with grape by-products did not significantly affect the nutritional properties of the chicken muscle tissue. However, colorimetric evaluation revealed significant changes: Lightness (L*) significantly increased in all treated groups compared to the control, with the greatest increase observed at the highest percentage of meal supplementation. Conversely, redness (a*) significantly decreased in all treatments compared to the control, particularly at the highest percentage of meal, while no significant change was observed for yellowness (b*). These results are summarized in Table 1.

### 3.2. Evaluation of Oxidative Stress

**Figure 1:**
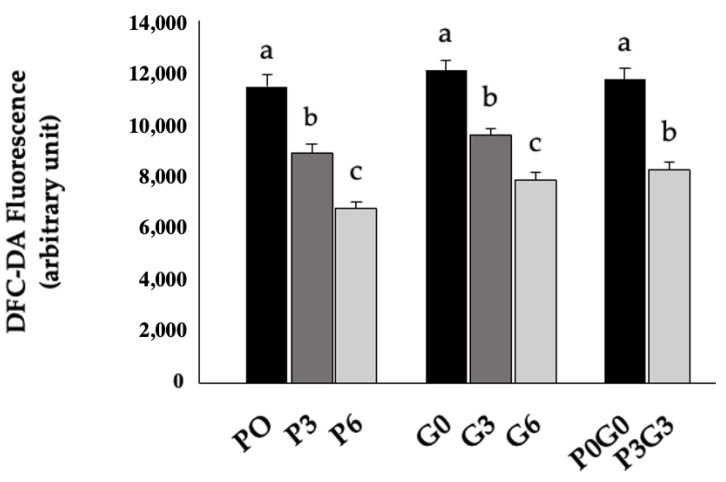
ROS production in muscle tissue from groups fed PMF (P0, P3 and P6), GSF (G0, G3 and G6), and a mixture of the two (P0G0 and P3G3) at different percentages of inclusion in the diet. Data are expressed as mean ± SEM. Bars with different letters (a–c) within the same group are significantly different for *p-*value *< 0.05*.

Muscle ROS levels were significantly reduced in all dietary treatment groups compared to controls. The most pronounced decreases were observed specifically in the groups with the highest percentage of supplementation (P6, G6, and P3G3, *p* < 0.05). The most pronounced effect was observed in GP meal group P, where the differences were significant, with inhibitions of 25% and 42% for P3 and P6, respectively, compared to the control P0. Although less marked than in group P, the experimental groups G and PG also showed statistically significant reductions in ROS levels of 21%, 35%, and 30% for G3, G6, and P3G3, respectively, compared to their controls, G0 and P0G0. The results are detailed in Figure 1.

**Figure 2:**
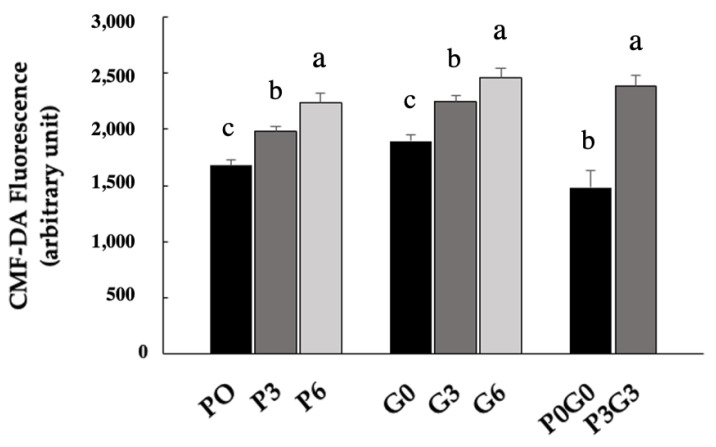
GSH in muscle tissue samples of broiler chickens fed PMF (P0, P3 and P6), GSF (G0, G3 and G6), and a mixture of the two (P0G0 and P3G3) at different percentages. The data are expressed as the mean ± SEM. Within the same group, bars with different letters (a–c) are significantly different with *p* < 0.05.

GSH levels were significantly increased in all dietary treatments compared to controls. In all of the experimental diets, GSH levels were significantly higher (*p* < 0.05), with the greatest increase observed in the blended treatment (P3G3), which recorded a 61% increase, compared to 33% and 2% observed for P6 and G6, respectively. The results are shown in Figure 2.

**Figure 3:**
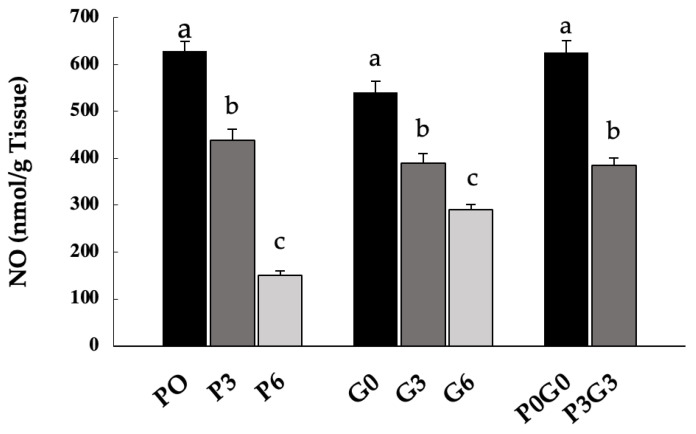
NO in muscle tissue samples of broiler chickens fed PMF (P0, P3 and P6), GSF (G0, G3 and G6), and a mixture of the two (P0G0 and P3G3) at different percentages. The data are expressed as the mean ± SEM. Within the same group, bars with different letters (a–c) are significantly different, with *p* < 0.05.

NO levels in muscle tissue (Figure 3) were significantly reduced in all experimental treatments compared to controls (*p* < 0.05), with the most pronounced reduction at the highest supplementation percentages (P6, G6, and P3G3). In particular, NO levels were significantly lower in the 6% PMF treatment (P6) compared to the 6% GSF treatment (G6, *p* < 0.05). Significant decreases were also observed when comparing different supplementation levels within the same type of meal (P3 vs. P6 and G3 vs. G6, *p* < 0.05), with the most pronounced effect at P6 and G6. At the highest concentrations, the most significant effect was exerted by P6, which caused a 76% reduction in NO, compared to 44% for G6 and 37% for P3G3.

### 3.3. Fatty Acid Composition

The fatty acid composition of muscle tissue (Table 2) is expressed as a relative percentage. Chemical analysis highlights that the supplementation significantly affected the acidic profile of muscle samples, proving a nutritionally desirable composition. Fatty acids detected were myristic acid, palmitic acid, stearic acid, palmitoleic acid, oleic acid, and linoleic acid, the percentages of which varied according to the diet. A significant decrease in saturated fatty acids (SFAs) and a significant increase in PUFAs in all experimental treatments were found compared to the control diet group, with the most pronounced effects observed at the highest percentages of supplementation (P6, G6, and P3G3, *p* < 0.05). Specifically, SFAs decreased in all treatments, with the greatest reduction observed in the P3G3 group. Monounsaturated fatty acids (MUFAs) showed a reduction in all treatments administered, with the exception of G3 (3% grape seed). PUFAs increased markedly in all supplemented treatments compared to the control, largely due to the high level of linoleic acid. The highest PUFA values were consistently observed in the high-dosage groups (P6, G6) and the combined P3G3 mix, confirming the direct benefit of the supplements.

### 3.4. Total Phenolic Content and Radical Scavenging Activity Assays

POV, TPC, DPPH, and ABTS^•+^ values are summarized in Table 3.

POV (meq O_2_/kg fat) was significantly decreased in all treatments compared to the control, with the most marked reduction observed at the highest percentages of by-product administration. TPC increased significantly in all treatments compared to the control, with the greatest increase observed with the co-administration of the flours (P3G3), reaching 4.21 ± 0.12 mg GAEx g^−1^. Similarly, radical scavenging activity (DPPH and ABTS^•+^) values increased in all treatments. The anti-radical activity was highest in the P3G3 group, reaching 7.822 mmolTEAC/100 g for the DPPH assay and 5.630 mmolTEAC/100 g for the ABTS assay. Significant increases were also observed in P6 (6.888 mmolTEAC/100 g) and G6 (6.459 mmolTEAC/100 g). P3G showed the most enhancement of both the total phenolic content and the radical scavenging capacity, leading to a chicken muscle product with antioxidant potential.

## 4. Discussion

The marked growth in poultry production, though driven by efficiency, requires the integration of high-value-added natural antioxidants into feed, while adhering to the stringent principles of the circular economy that promote the valorization of agri-food residues, such as WBPs. In this context, our results demonstrated that the inclusion of WBPs in broiler chicken diets did not alter the fundamental macromolecular composition of the meat (protein, lipids, minerals), aligning with studies confirming their basic nutritional value without adverse effects at tested inclusion rates [36,37]. However, in agreement with previous studies [38,39,40,41,42,43,44], positive qualitative modifications were evident, particularly regarding meat color: the addition of both pomace and seeds caused a significant linear reduction in redness (a*) and an increase in lightness (L*). This chromatic shift is considered to be a result of the transfer of WBP pigments, such as anthocyanins [45], and is commercially advantageous as consumers tend to prefer light-red meat.

The primary benefit of WBPs lies in their antioxidant capacity, validated by the reductions in POV, especially at higher doses. This is crucial since lipid peroxidation negatively impacts the shelf life and sensory qualities of meat, potentially generating harmful side compounds for human health [12]. The sensitivity to oxidation of meat is controlled by the balance between unsaturated fatty acids and antioxidants; our data strongly support the hypothesis that phenolic compounds transferred from WBPs actively stabilize muscle lepidic components. The antioxidant effect was confirmed systematically: the decrease in ROS levels at 42 days, consistent with parallel plasma analyses [23], suggests protection against oxidative damage. This effect correlates with the absorption of key polyphenols such as catechins and proanthocyanidins [46]. Concurrently, an increase in GSH levels indicates that WBP flavonoids stimulate the animal’s endogenous enzymatic antioxidant response [47]. The literature reports benefits for animal health, suggested by the decrease in NO levels, a significant indicator of systemic inflammation, particularly in groups treated with high pomace concentrations [48].

Among desirable effects from supplementation in this trial, striking evidence emerged as to a different fatty acid profile, where a marked reduction in SFA and a significant increase in PUFA concentrations were observed in comparison to the control diet group [18,19]. The highest PUFA percentage (15.6%) was recorded in the P3G3 combined diet group, reflecting the high content of linoleic acid in grape seeds [38]. This modification of the lipid profile enhances the nutritional value of the meat and contributes to its greater stability. TPC and radical scavenging activities (DPPH and ABTS^•+^) further reinforced that maximum efficacy is achieved with the synergistic mixture, where the benefits of liposoluble components (seeds) combine with hydrosoluble/bound phenolics (pomace) [16]. Ultimately, the targeted inclusion of WBPs serves as a dual strategy, addressing an environmental concern while offering an effective solution for improving the nutritional quality and shelf life of chicken meat.

## 5. Conclusions

The results obtained in this study convincingly support the integration of flours derived from WBPs into the poultry food chain as a source of natural, functional antioxidants.

The results pointed out that a combined mixture of grape pomace and grape seed meal significantly improved the oxidative stability of the meat, evidenced by the reduction in ROS and NO levels (suggesting an anti-inflammatory and cellular protective effect) and an increase in GSH.

Crucially, the inclusion of WBPs led to different fatty acid profiles of chicken meat, increasing PUFA proportions and meeting the growing consumer demand for healthier meat products with improved nutritional value. Such nutritional modulation, coupled with the increased total phenolic content and antioxidant characteristics of muscle, positions WBPs as valuable natural substitutes for conventional synthetic antioxidants (such as BHA, BHT, and ethoxyquin).

The influence of residual pigments on meat color (increased lightness L* and modification of redness a*) suggests desirable meat benefits and potential extension of shelf life. In conclusion, key findings of this study clearly recommend the use of WBP-derived flours as an effective strategy to improve the quality and shelf life of chicken meat, while offering a dual-advantage solution that reduces reliance on synthetic additives and actively promotes the principles of the circular economy in animal husbandry.

## Figures and Tables

**Table 1:** Proximal composition (g/100 g), colorimetric parameters, physical traits, and oxidation status of muscle tissue of broiler chickens fed pomace flour (P0, P3 and P6), grape seed flour (G0, G3 and G6), and a mix of the two (P0G0 and P3G3) at different percentages.

	P0	P3	P6	*p-*Value	SEM	G0	G3	G6	*p-*Value	SEM	P0G0	P3G3	*p-*Value	SEM
Moisture	74.43	74.17	74.42	0.705	3.02	74.76	74.58	74.67	0.658	3.11	74.39	74.25	0.839	3.09
Protein	22.31	22.47	22.18	0.484	1.43	22.06	21.99	22.19	0.563	1.51	22.59	21.94	0.785	1.01
Fat	1.78	1.91	1.84	0.292	0.10	1.71	1.70	1.80	0.389	0.09	1.71	1.89	0.662	0.12
Ash	1.32	1.34	1.48	0.436	0.09	1.32	1.32	1.29	0.865	0.08	1.34	1.43	0.357	0.11
Warner–Bratzler shear force, kg/cm^2^	2.96	2.91	3.05	0.461	0.17	3.02	2.97	3.09	0.512	0.20	2.97	3.03	0.912	0.13
L*, lightness	49.9 ^b^	52.4 ^ab^	59.7 ^a^	0.035	2.86	50.1 ^c^	55.4 ^b^	60.1 ^a^	0.023	2.75	50.9 ^b^	56.4 ^a^	0.043	2.52
a*, redness	4.2 ^a^	3.9 ^ab^	3.7 ^b^	0.039	0.21	4.1 ^a^	4.0 ^a^	3.6 ^b^	0.043	0.18	4.3 ^a^	3.7 ^b^	0.021	0.16
b*, yellowness	3.2	3.3	3.1	0.263	0.19	3.5	3.3	3.4	0.125	0.20	3.2	3.3	0.656	0.15

a–c: means with different letters (a–c) in a row within a trial are significantly different for *p-*value ≤ 0.05. SEM = standard error of mean.

**Table 2:** Fatty acid composition expressed as a relative percentage of muscle tissue of broiler chickens fed pomace flour (P0, P3 and P6), grape seed flour (G0, G3 and G6), and a mix of the two (P0G0 and P3G3) at different percentages.

Fatty Acid (rel. %)	P0	P3	P6	*p-*Value	SEM	G0	G3	G6	*p-*Value	SEM	P0G0	P3G3	*p-*Value	SEM
Myristic	5.69 ^a^	4.65 ^b^	4.36 ^b^	0.003	0.27	5.01 ^a^	3.89 ^b^	3.09 ^b^	0.005	0.38	5.76 ^a^	3.54 ^b^	0.002	0.51
Myristoleic	0.35	0.46	0.46	0.845	0.09	0.43	0.47	0.51	0.657	0.04	0.48	0.53	0.315	0.02
Pentadecanoic	0.51	0.49	0.43	0.053	0.02	0.50	0.49	0.41	0.063	0.02	0.52 ^a^	0.32 ^b^	<0.0001	0.05
Palmitic	30.68 ^a^	27.19 ^ab^	22.80 ^b^	0.010	1.58	29.50 ^a^	29.59 ^a^	23.62 ^b^	0.019	1.42	30.49 ^a^	18.08 ^b^	0.007	2.99
Palmitoleic	2.95	2.90	2.92	0.943	0.07	2.89	2.76	2.34	0.166	0.15	3.00	2.92	0.395	0.04
Heptadecenoic	0.81	0.70	0.70	0.185	0.03	0.80 ^a^	0.55 ^b^	0.43 ^c^	0.0001	0.07	0.84 ^a^	0.48 ^b^	0.001	0.08
Stearic	17.39	15.61	15.49	0.496	0.83	17.63	12.46	11.45	0.086	1.57	17.25 ^a^	14.87 ^b^	0.029	0.62
Oleic	32.88	35.58	37.03	0.320	1.33	32.97 ^b^	36.45 ^ab^	42.42 ^a^	0.045	2.11	32.77 ^b^	45.89 ^a^	0.0002	2.97
Linoleic	8.62 ^b^	12.24 ^ab^	14.92 ^a^	0.006	1.24	8.94 ^c^	12.49 ^b^	14.82 ^a^	0.0002	1.08	8.99 ^b^	15.48 ^a^	0.0005	1.48
α-Linolenic	0.11	0.15	0.15	0.177	0.01	0.11	0.12	0.15	0.314	0.01	0.11 ^b^	0.13 ^a^	0.029	0.01
%SFAs	55.08 ^a^	48.65 ^ab^	43.77 ^b^	0.018	2.34	54.88 ^a^	46.98 ^b^	39.00 ^c^	<0.0001	2.86	54.52 ^a^	37.30 ^b^	0.0004	3.91
%MUFAs	36.18	38.96	40.41	0.318	1.35	36.37 ^b^	39.68 ^ab^	45.26 ^a^	0.0370	1.95	36.97 ^b^	49.33 ^a^	<0.0001	2.79
%PUFAs	8.73 ^b^	12.39 ^ab^	15.08 ^a^	0.006	1.25	8.06 ^c^	12.61 ^b^	14.98 ^a^	<0.0001	1.27	8.80 ^b^	15.62 ^a^	0.0005	1.55

%SFAs = Σ saturated fatty acids/total fatty acids; %MUFAs = Σ monounsaturated fatty acids/total fatty acids. %PUFAs = Σ polyunsaturated fatty acids/total fatty acids. a–c: means with different letters (a–c) in a row within a trial are significantly different for *p-*value ≤ 0.05. SEM = standard error of mean.

**Table 3:** Peroxide value (POV, meq O_2_ kg/fat), total phenolic content (TPC, mgGAE/g), and radical scavenging activity (DPPH and ABTS^•+^, mmolTEAC/100 g) of muscle tissue of broiler chickens fed PMF (P0, P3 and P6), GSF (G0, G3 and G6), and a mixture of the two (P0G0 and P3G3) at different percentages.

	P0	P3	P6	*p-*Value	SEM	G0	G3	G6	*p-*Value	SEM	P0G0	P3G3	*p-*Value	SEM
POV meq O_2_/kg fat	1.95 ^a^	1.27 ^b^	0.96 ^c^	0.005	0.07	1.97 ^a^	1.09 ^b^	0.91 ^b^	0.009	0.09	2.04 ^a^	1.01 ^b^	0.011	0.12
TPC mgGAE/g	0.30 ^b^	2.78 ^a^	3.17 ^a^	<0.0001	0.46	0.25 ^b^	2.94 ^a^	3.48 ^a^	0.001	0.52	0.28 ^b^	4.21 ^a^	<0.0001	0.80
DPPH mmolTEAC/100 g	2.48 ^c^	4.28 ^b^	6.89 ^a^	0.001	0.67	2.49 ^b^	5.21 ^a^	6.46 ^a^	0.001	0.61	2.26 ^b^	7.82 ^a^	0.0002	1.25
ABTS mmolTEAC/100 g	1.94 ^b^	5.97 ^a^	6.36 ^a^	0.001	0.74	1.65 ^b^	4.02 ^a^	4.76 ^a^	0.009	0.53	1.89 ^b^	5.63 ^a^	0.002	0.87

a–c: means with different letters (a–c) in a row within a trial are significantly different for *p*-value ≤ 0.05. SEM = standard error of mean.

## Data Availability

Data are not shared for privacy reasons; please contact the authors with requests.

## Supplementary Materials

The following supporting information can be downloaded at: https://www.mdpi.com/article/10.3390/ani16020280/s1, Tables S1–S4: Chemical characterization of grape by-products and composition of diets utilized in the experiments.
