# Supplementary material for: Supplemented Feed for Broiler Chickens: The Influence of Red Grape Pomace and Grape Seed Flours on Meat Characteristics
Source: Animals (Basel). 2026 Jan 16;16(2):280. doi: 10.3390/ani16020280 (PMC12838070; doi:10.3390/ani16020280)
Supplement: Supplementary file 1 [file animals-16-00280-s001.zip › animals-4034139-supplementary.PDF]

Table1. Chemical characterization of pomace and grape seed flour.

	**Pomace**	**Grape seed**	
Dry matter (DM %)	92.31	95.55	(AOAC method 934.01)
Crude Protein	12.64	14.69	(AOAC method 2001.11)
Ether extract	4.69	3.19	(AOAC method 920.39)
aNDFom	33.49	55.35	(AOAC method 2002.04)
Ash	7.48	3.76	(AOAC method 942.05)
Non-structural carbohydrates	41.70	23.01	

Table 2. Composition of diet supplemented using grape pomace flour. All nutrients are expressed as % feed except selenium in ppm, vitamins in International Units (IU) and Amen in kcal/kg

	P0				*P3*				*P6*			
Ingr./Nutr	*Pre-starter*	*Starter*	*Grower*	*Finisher*	*Pre-starter*	*Starter*	*Grower*	*Finisher*	*Pre-starter*	*Starter*	*Grower*	*Finisher*
Corn	46	49	54	56	46.9	49	55.2	57.507	47.9845	48.5	55.3	56.482
Soybean Meal	36.875	33.15	28.7	2.,175	34.9	34	28.1	25.8	34.725	34.2	27.8	26.25
Fat and Oil	3.8	5	5.75	5.6	3.9	5	5.95	6	3.85	5.4	5.85	5.85
Grape Pomace	***0***	***0***	***0***	***0***	***3***	***3***	***3***	***3***	***6***	***6***	***6***	***6***
Other ingredients	13.3	12.9	11.6	12.2	11.3	9.0	7.7	7.7	7.4	5.9	5.1	5.4
VOLUME	100	100	100	100	100	100	100	100	100	100	100	100
DM	88.6	88.5	88.5	88.5	88.8	88.6	88.7	88.7	88.9	88.8	88.8	88.8
AMEN	2980	3075	3168	3189	2979	3075	3182	3195	2980	3075	3180	3195
CP	23.1	20.8	18.8	18.0	22.8	20.9	18.9	18.1	22.8	20.9	18.8	18.1
CF	3.6	3.7	3.4	3.5	3.5	4.0	3.6	3.7	3.9	4.5	4.0	3.9
EE	6.1	7.2	8.2	8.2	6.1	7.3	8.4	8.3	6.1	7.7	8.6	8.6
ASH	7.0	6.3	5.5	5.2	6.9	6.2	5.4	5.2	6.9	6.2	5.4	5.2
#D LYS	1.3	1.2	1.0	1.0	1.3	1.2	1.0	1.0	1.3	1.2	1.0	1.0
#D MET	0.7	0.6	0.5	0.5	0.6	0.6	0.5	0.5	0.6	0.6	0.5	0.5
CA TOT	1.0	0.9	0.8	0.7	1.0	0.9	0.8	0.7	1.0	0.9	0.8	0.7
P TOT	0.7	0.6	0.5	0.5	0.7	0.6	0.5	0.5	0.7	0.6	0.5	0.5
SELENIUM	0.3	0.25	0.25	0.25	0.3	0.25	0.25	0.25	0.3	0.25	0.25	0.25
VIT A	12000	10000	10000	10000	12000	10000	10000	10000	12000	10000	10000	10000
VIT D	4950	4125	4125	4125	4950	4125	4125	4125	4950	4125	4125	4125
VIT E	84	70	70	70	84	70	70	70	84	70	70	70

Table 3. Composition of diet supplemented using grape seed flours. All nutrients are expressed as % feed except selenium in ppm, vitamins in International Units (IU) and Amen in kcal/kg

	G0				*G3*				*G3*			
Ingr./Nutr	*Pre-starter*	*Starter*	*Grower*	*Finisher*	*Pre-starter*	*Starter*	*Grower*	*Finisher*	*Pre-starter*	*Starter*	*Grower*	*Finisher*
Corn	46.8895	49.2145	54.162	53.027	44.5295	50.4095	55.07	54.47	44.317	50.7995	55.765	54.6
Soybean Meal	37.95	32.65	28.325	25.625	36.9	32.275	28.8	27.075	38.225	33.425	29.925	28.17
Fat and Oil	3.7	4.1	5.6	6.2	4.47	4.3	5.7	6.7	475	4.5	5.85	7
Grape Seed	**0**	**0**	**0**	**0**	**3**	**3**	**3**	**3**	**6**	**6**	**6**	**6**
Other ingredients	11.4605	14.0355	11.913	15.148	11.1005	10.0155	7.43	8.755	-463.542	5.2755	2.46	4.23
VOLUME	100	100	100	100	100	100	100	100	100	100	100	100
DM	89.4	89.2	89.2	89.1	89.6	89.4	89.4	89.5	89.8	89.5	89.5	89.7
AMEN	2991	3080	3180	3204	2991	3080	3179	3210	2990	3079	3179	3210
CP	23.0	21.1	19.1	18.3	23.2	21.1	19.1	18.3	23.3	21.1	19.2	18.4
CF	2.9	2.9	2.8	2.9	4.2	4.1	3.9	4.1	4.8	4.7	4.6	4.8
EE	6.2	6.7	8.2	8.8	6.9	6.8	8.2	9.2	7.2	7.0	8.4	9.4
ASH	6.5	5.7	5.2	4.7	6.4	5.7	5.1	4.7	6.3	5.6	5.1	4.7
#D LYS	1.29	1.15	1.02	0.97	1.29	1.15	1.02	0.96	1.29	1.15	1.02	0.96
#D MET	0.67	0.61	0.55	0.51	0.68	0.61	0.56	0.53	0.67	0.61	0.57	0.52
CA TOT	0.96	0.89	0.83	0.75	0.96	0.90	0.82	0.74	0.97	0.90	0.82	0.74
P TOT	0.72	0.59	0.54	0.49	0.71	0.58	0.53	0.48	0.70	0.57	0.52	0.47
SELENIUM	0.30	0.25	0.25	0.25	0.30	0.25	0.25	0.25	0.30	0.25	0.25	0.25
VIT A	12000	10000	10000	10000	12000	10000	10000	10000	12000	10000	10000	10000
VIT D	4950	4125	4125	4125	4950	5000	4125	4125	4950	5000	4125	4125
VIT E	84	70	70	70	84	70	70	70	84	70	70	70

Table 4. Composition of diet supplemented using grape pomace and grape seed flours. All nutrients are expressed as % feed except selenium in ppm, vitamins in International Units (IU) and Amen in kcal/kg

	P0G0				*P3G3*			
Ingr./Nutr	*Pre-starter*	*Starter*	*Grower*	*Finisher*	*Pre-starter*	*Starter*	*Grower*	*Finisher*
Corn	43.42	48.04	51.86	53.945	42.695	50.465	50.08	54.392
Soybean Meal	37.35	33.725	28.65	26.2	40.7	33.8	29.925	29.45
Fat and Oil	4.4	5.05	6.25	6.4	5.3	4.9	5.9	5.9
Grape Pomace	***0***	***0***	***0***	***0***	***3***	***3***	***3***	***3***
Grape Seed	***0***	***0***	***0***	***0***	***3***	***3***	***3***	***3***
Other Ingredients	14.83	13.185	13.24	13.455	5.305	4.835	8.095	4.258
VOLUME	100	100	100	100	100	100	100	100
DM	89.5	89.3	89.3	89.2	89.9	89.7	89.4	89.3
AMEN	2990	3079	3180	3209	2990	3081	3131	3160
CP	23.4	21.2	19.1	18.2	23.5	21.2	20.4	19.2
CF	3.5	3.4	3.3	3.3	4.9	4.6	5.0	4.9
EE	6.6	7.2	8.5	8.6	7.4	7.4	8.5	8.5
ASH	6.4	5.7	5.1	4.8	6.5	5.6	4.8	4.5
#D LYS	1.3	1.2	1.0	1.0	1.3	1.2	1.1	1.0
#D MET	0.7	0.6	0.6	0.5	0.7	0.6	0.6	0.6
CA TOT	1.0	0.9	0.8	0.7	1.0	0.9	0.6	0.6
SELENIUM	0.3	0.3	0.3	0.3	0.3	0.3	0.3	0.3
P TOT	0.7	0.6	0.5	0.5	0.7	0.6	0.5	0.4
VIT A	12000	10000	10000	10000	12000	10000	10000	10000
VIT D	4950	5000	4125	4125	4950	5000	4125	4125
VIT E	84	70	70	70	84	70	70	70
